# Supplementary material for: Multi-omic profiling to assess the effect of iron starvation in Streptococcus pneumoniae TIGR4
Source: PeerJ. 2018 Jun 13;6:e4966. doi: 10.7717/peerj.4966 (PMC6004102; doi:10.7717/peerj.4966)
Supplement: Supplemental Information 1 — The file contains five supplementary figures, one supplementary table and one supplementary dataset. [file peerj-06-4966-s001.pdf]

Multi-omic profiling to assess the effect of iron starvation in *Streptococcus pneumoniae* TIGR4

Irene Jiménez-Munguía[1], Mónica Calderón-Santiago[2], Antonio Rodríguez-Franco[1], Feliciano Priego-Capote[2], Manuel J. Rodríguez-Ortega[1] *.

[1]Departamento de Bioquímica y Biología Molecular, Universidad de Córdoba; Campus de Excelencia Internacional CeiA3.

[2]Departamento de Química Analítica, Universidad de Córdoba; Campus de Excelencia Internacional CeiA3, Córdoba (Spain).

*Corresponding author:

Manuel J. Rodríguez-Ortega

Address: Departamento de Bioquímica y Biología Molecular, Edificio "Severo Ochoa" planta baja, Campus de Rabanales, Universidad de Córdoba. 14071 Córdoba, Spain.

Tel: +34 957 218519

Fax: +34 957 218856

e-mail: mjrodriguez@uco.es

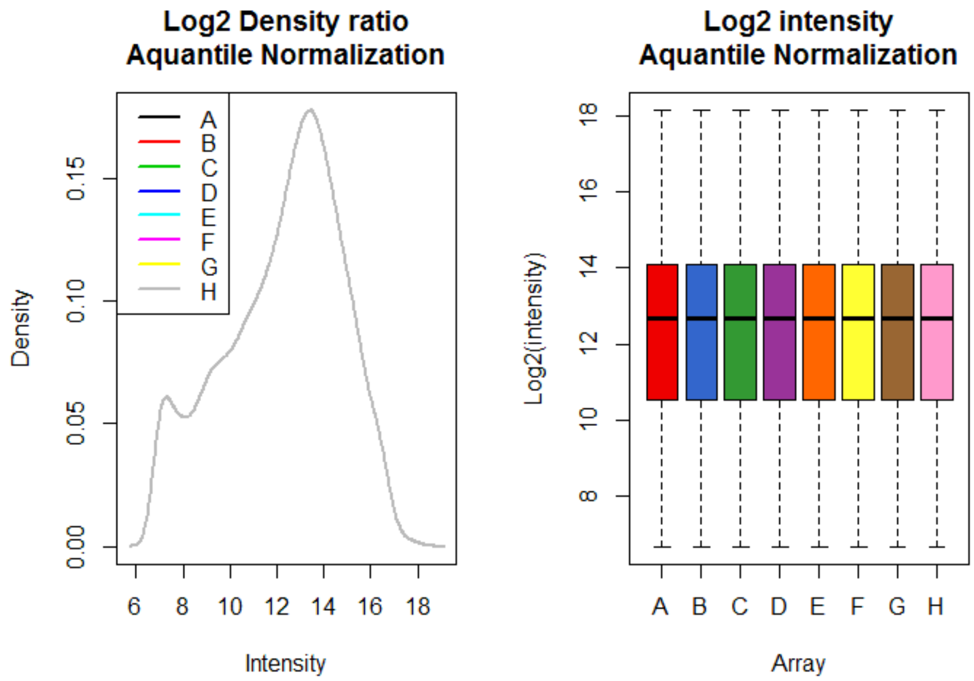

Fig. S1. Normalized data of the DNA microarray. Left panel: Density of intensity signal graph after normalization. Right panel: Intensity graph after normalization. This included two steps: a within and a between normalization with the Limma package as described in Material and Methods.

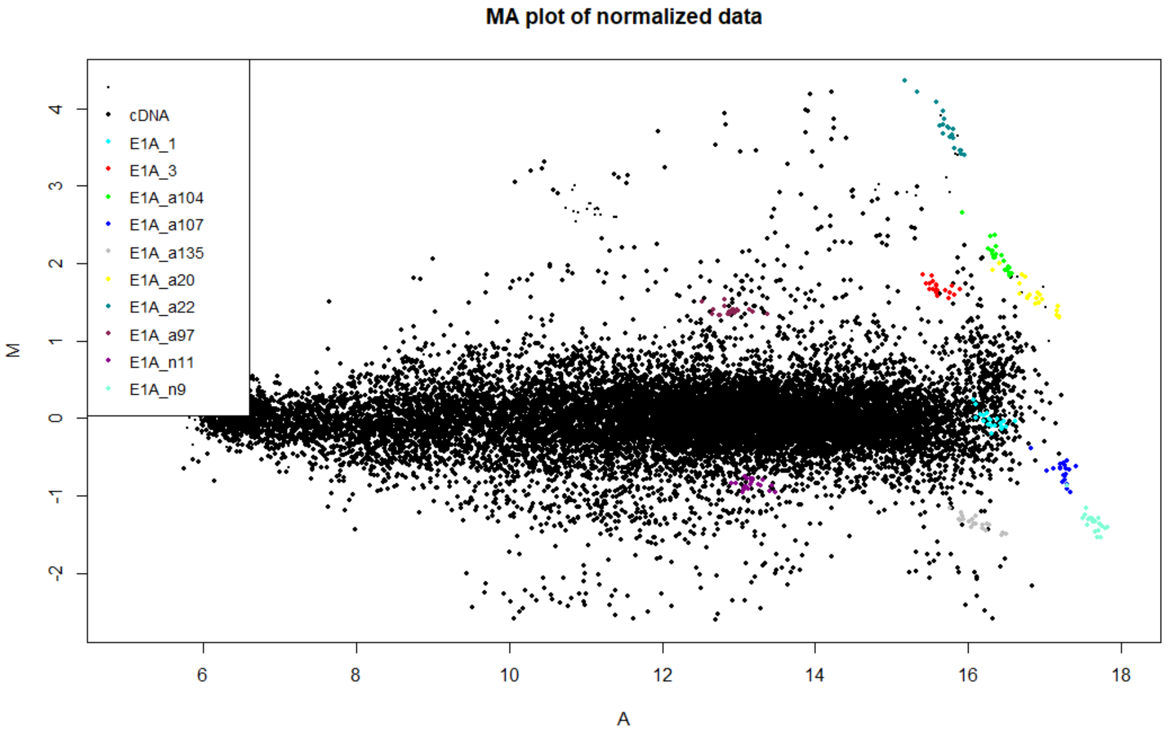

Fig. S2. MA plot of normalized data. Data obtained from microarrays were background subtracted, then normalized according the within-loess and the between-aquantile, and finally was averaged. The black spots correspond to cDNA. The color spots are the Spike-in controls used in the microarray.

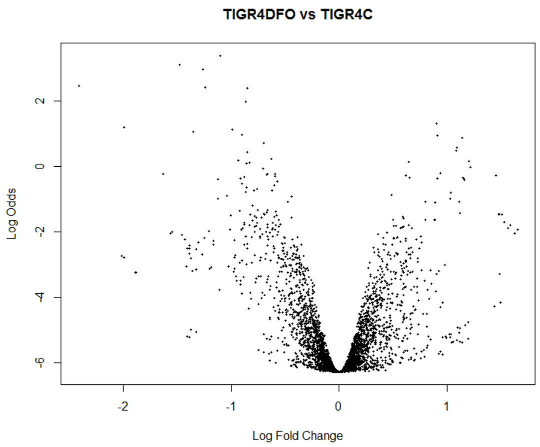

Fig. S3. Volcano plot corresponding to the differentially expressed genes in *Streptococcus pneumoniae* TIGR4 subject to iron starvation after deferoxamine (DFO) treatment.

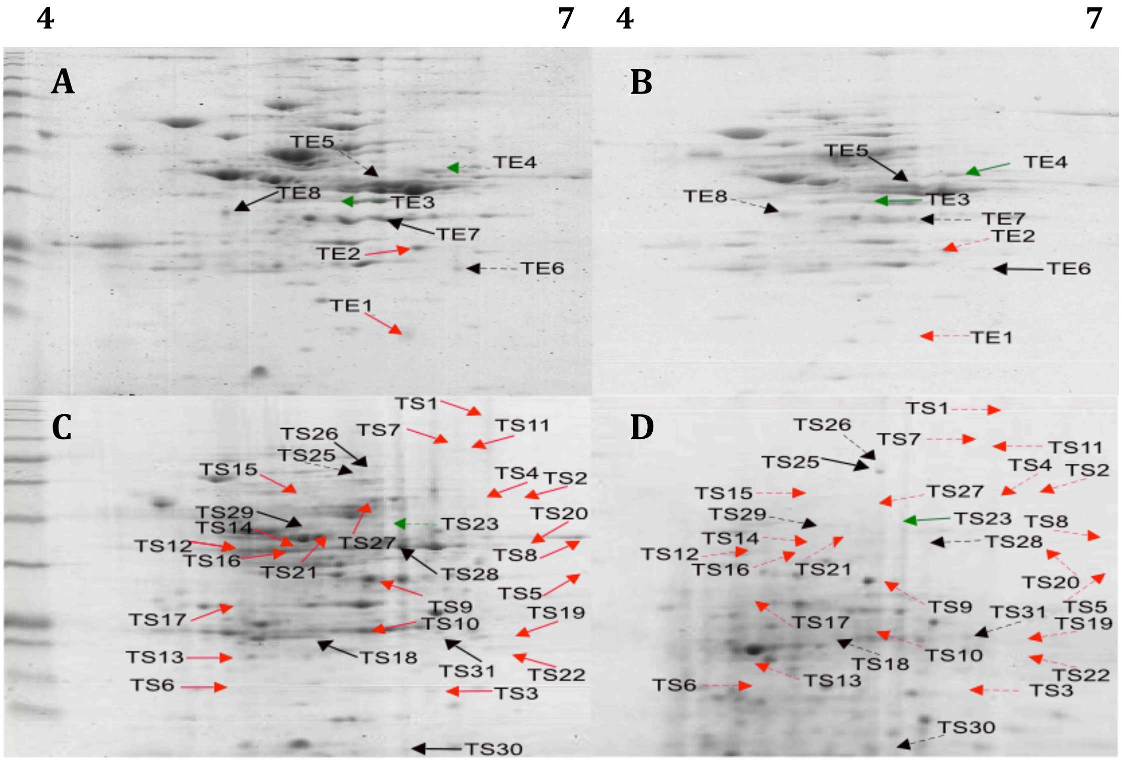

Fig. S4. 2-DE protein profiles of *Streptococcus pneumoniae* TIGR4. A) Control total extract; B) deferoxamine (DFO)-treated total extract; C) control secretome; and D) DFO-treated secretome. Differential proteins are indicated by arrows: Red, spots absent in the DFO-treated samples; Green, spots only present in the DFO-treated samples; and Black, proteins present in both control and treatments, but with increased abundance (>2 fold-change, solid line) or with decreased abundance (<0.5 fold-change, dotted line) in the DFO-treated samples compared to the control.

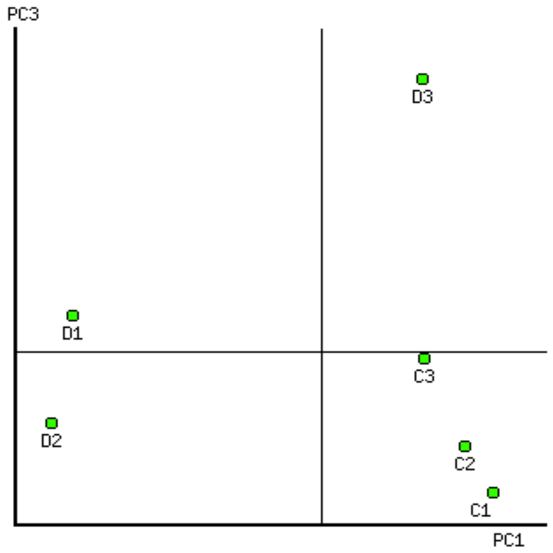

Fig. S5. Principal component analysis plot of differentially abundant metabolites in *Streptococcus pneumoniae* TIGR4. Dots C1-C3 represent the three biological replicates of the control and D1-D3 the three biological replicates of the deferoxamine (DFO) treatment.

Table S1. Microarray validation by RT-qPCR

	Log_2 FC	
Locus	RT-qPCR	Microarray
SP_0324	-0.39	-0.99
SP_1027	0.28	0.97
SP_0641	0.31	1.03
SP_0176	0.67	1.19
SP_2196	0.25	0.61
SP_1871	0.55	1.51
SP_0981	0.4	0.95
SP_1894	-0.21	-0.65
SP_1591	0.58	0.73
SP_1466	-0.48	-1.45
SP_1857	0.83	1.44
SP_2233	-1.05	-2.05
SP_0427	-0.86	-1.37
SP_0426	-0.61	-1.26
SP_0390	0.23	0.43
SP_2239	1.13	1.64

Supplementary Dataset: Differentially expressed genes in Streptococcus pneumoniae TIGR4 after deferoxamine treatment.

No.	Accession (GI)	Locus	Description	Location	Log_2FC	*p*-value	ID OPERON
1	15899975	SP_0029	Uncharacterized protein	Cytoplasmic	0,46	0,04	38470
2	15899980	SP_0034	UPF0324 membrane protein SP_0034	Membrane	0,53	0,02	1446351
3	15900028	SP_0084	Histidine kinase	Membrane	0,32	0,04	38480
4	15900059	SP_0117	Pneumococcal surface protein A	Membrane	0,8	0,02	1446388
5	15900111	SP_0174	Uncharacterized protein	Cytoplasmic	1	0,01	1446404
6	15900112	SP_0175	6,7-dimethyl-8-ribityllumazine synthase	Cytoplasmic	1,13	0	38501
7	15900113	SP_0176	Riboflavin biosynthesis protein RibBA	Cytoplasmic	1,19	0,01	38501
8	15900114	SP_0177	Riboflavin synthase, alpha subunit	Cytoplasmic	1,13	0,01	38501
9	15900115	SP_0178	Riboflavin biosynthesis protein RibD	Cytoplasmic	1,13	0,01	38501
10	15900138	SP_0202	Anaerobic ribonucleoside-triphosphate reductase	Cytoplasmic	0,61	0,01	38508
11	15900139	SP_0203	Uncharacterized protein	Cytoplasmic	0,43	0,03	38508
12	15900140	SP_0204	Acetyltransferase, GNAT family	Cytoplasmic	0,66	0	38509
13	15900141	SP_0205	Anaerobic ribonucleoside-triphosphate reductase-activating protein	Cytoplasmic	0,64	0,01	38509
14	15900142	SP_0206	Uncharacterized protein	Cytoplasmic	0,61	0,01	38509
15	15900143	SP_0207	Conserved domain protein	Cytoplasmic	0,52	0,01	38509
16	15900215	SP_0281	Aminopeptidase C	Cytoplasmic	0,92	0,01	1446426
17	15900298	SP_0375	6-phosphogluconate dehydrogenase, decarboxylating	Cytoplasmic	0,41	0,02	38538
18	15900308	SP_0385	Uncharacterized protein	Membrane	0,44	0,05	38541
19	15900309	SP_0386	Putative sensor histidine kinase	Membrane	0,6	0,02	38541
20	15900312	SP_0390	Choline binding protein G	Cytoplasmic	0,43	0,03	38542
21	15900429	SP_0515	Heat-inducible transcription repressor HrcA	Cytoplasmic	0,84	0,04	38567
22	15900430	SP_0516	Protein GrpE	Cytoplasmic	0,8	0,04	38567
23	15900432	SP_0518	Uncharacterized protein	Cytoplasmic	1,05	0,02	38568
24	15900507	SP_0599	Transmembrane protein Vexp1	Membrane	0,79	0,01	38588
25	15900508	SP_0600	ABC transporter, ATP-binding protein Vexp2	Cytoplasmic	0,8	0,01	38588
26	15900509	SP_0601	Transmembrane protein Vexp3	Membrane	0,8	0,01	38588
27	15900511	SP_0603	DNA-binding response regulator VncR	Cytoplasmic	0,66	0,01	38589
28	15900512	SP_0604	Histidine kinase	Membrane	0,53	0,02	38589
29	15900513	SP_0605	Fructose-bisphosphate aldolase	Cytoplasmic	0,58	0,02	1446528
30	15900524	SP_0616	Beta-lactam resistance factor	Cytoplasmic	0,49	0,02	38592
31	15900525	SP_0617	Conserved domain protein	Membrane	0,56	0,02	38593
32	15900530	SP_0622	Nitroreductase family protein	Cytoplasmic	0,89	0,02	38594
33	15900531	SP_0623	Dipeptidase	Cytoplasmic	0,52	0,02	38594
34	15900532	SP_0624	Uncharacterized protein	Cytoplasmic	0,43	0,02	38594
35	15900534	SP_0627	Uncharacterized protein	Cytoplasmic	0,59	0,03	38595
36	15900535	SP_0628	HIT family protein	Cytoplasmic	0,71	0,01	38595
37	15900547	SP_0641	Serine protease, subtilase family	Cell Wall	1,03	0,01	1446538
38	15900561	SP_0660	Peptide methionine sulfoxide reductase MsrA/MsrB 2 [Includes: Peptide methionine sulfoxide reductase MsrA	Cytoplasmic	0,46	0,04	1446542
39	15900562	SP_0661	DNA-binding response regulator	Cytoplasmic	0,6	0,02	38600
40	15900563	SP_0662	Putative sensor histidine kinase	Membrane	0,5	0,02	38600
41	15900677	SP_0784	Glutathione reductase	Cytoplasmic	0,75	0,03	1446583
42	15900691	SP_0798	Transcriptional regulatory protein CiaR	Cytoplasmic	0,71	0,03	38630
43	15900751	SP_0868	Uncharacterized protein	Cytoplasmic	0,44	0,03	38642
44	15900752	SP_0869	Cysteine desulfurase	Cytoplasmic	0,4	0,04	38642
45	15900782	SP_0901	Uncharacterized protein	Cytoplasmic	0,62	0,02	38651
46	15900786	SP_0905	Uncharacterized protein	Membrane	0,52	0,03	38652
47	15900858	SP_0981	Foldase protein PrsA	Lipoprotein	0,95	0,01	38668
48	15900868	SP_0993	Exonuclease	Cytoplasmic	0,3	0,04	38671
49	15900872	SP_0999	Cytochrome c-type biogenesis protein CcdA	Membrane	0,6	0,01	38672
50	15900873	SP_1000	Thioredoxin family protein	Lipoprotein	0,47	0,03	38672
51	15900898	SP_1027	Uncharacterized protein	Secretory	0,97	0,03	38676
52	15900963	SP_1095	Ribose-phosphate pyrophosphokinase 2	Cytoplasmic	0,41	0,03	38687
53	15901082	SP_1220	L-lactate dehydrogenase	Cytoplasmic	0,64	0,04	1446720
54	15901144	SP_1284	LemA protein	Membrane	0,36	0,03	38728
55	15901192	SP_1338	Uncharacterized protein	Membrane	0,72	0,02	1446757
56	15901194	SP_1340	Uncharacterized protein	Membrane	0,66	0,02	38738
57	15901195	SP_1341	ABC transporter, ATP-binding protein	Cytoplasmic	0,75	0,02	38738
58	15901196	SP_1342	Drug efflux ABC transporter, ATP-binding/permease protein	Membrane	0,82	0,02	38738
59	15901197	SP_1343	Prolyl oligopeptidase family protein	Cytoplasmic	0,95	0,01	38738
60	15901198	SP_1344	Uncharacterized protein	Cytoplasmic	0,88	0,02	38738
61	15901324	SP_1474	Glycine--tRNA ligase beta subunit	Cytoplasmic	0,42	0,04	38768
62	15901351	SP_1504	TPR domain protein	Cytoplasmic	0,29	0,04	38775
63	15901356	SP_1509	ATP synthase gamma chain	Cytoplasmic	0,47	0,03	38776
64	15901357	SP_1510	ATP synthase subunit alpha	Cytoplasmic	0,36	0,04	38776
65	15901358	SP_1511	ATP synthase subunit delta	Cytoplasmic	0,49	0,02	38776
66	15901359	SP_1512	ATP synthase subunit b	Membrane	0,48	0,03	38776
67	15901360	SP_1513	ATP synthase subunit a	Membrane	0,36	0,03	38776
68	15901409	SP_1566	UPF0042 nucleotide-binding protein SP_1566	Cytoplasmic	0,34	0,04	38789
69	15901412	SP_1569	ATP-dependent Clp protease ATP-binding subunit ClpX	Cytoplasmic	0,46	0,02	38789
70	15901430	SP_1588	Oxidoreductase, pyridine nucleotide-disulfide, class I	Cytoplasmic	0,89	0,02	1446822
71	15901433	SP_1591	Proline dipeptidase	Cytoplasmic	0,73	0,02	38795
72	15901434	SP_1592	Conserved domain protein	Membrane	0,67	0,02	38795
73	15901484	SP_1648	Manganese ABC transporter, ATP-binding protein	Cytoplasmic	0,67	0,02	38805
74	118090037	SP_1649	Putative manganese ABC transporter, permease protein	Membrane	0,72	0,01	38805
75	15901485	SP_1650	Manganese ABC transporter substrate-binding lipoprotein	Lipoprotein	0,7	0,03	38805
76	255964977	SP_1651	Probable thiol peroxidase	Cytoplasmic	0,53	0,03	1447025
77	15901497	SP_1662	YlmH protein	Cytoplasmic	0,42	0,01	38808
78	15901500	SP_1665	YlmE protein	Cytoplasmic	0,31	0,04	38808
79	15901504	SP_1669	MutT/nudix family protein	Cytoplasmic	0,49	0,03	38809
80	15901505	SP_1670	UDP-N-acetylmuramoyl-tripeptide--D-alanyl-D-alanine ligase	Cytoplasmic	0,47	0,02	38809
81	15901530	SP_1696	Uncharacterized protein	Cytoplasmic	0,86	0,05	1446861
82	15901549	SP_1715	ABC transporter, ATP-binding protein	Membrane	0,95	0,01	38820
83	15901603	SP_1774	Putative transcriptional regulator	Cytoplasmic	0,57	0,03	38833
84	15901604	SP_1775	Conserved domain protein	Cytoplasmic	0,67	0,02	38833
85	15901630	SP_1801	Uncharacterized protein	Membrane	0,73	0,01	1446883
86	15901631	SP_1802	Uncharacterized protein	Membrane	0,65	0,02	38839
87	15901632	SP_1803	Uncharacterized protein	Membrane	0,59	0,04	38839
88	15901633	SP_1804	Putative general stress protein 24	Cytoplasmic	0,67	0,03	38839
89	15901637	SP_1808	Putative type IV prepilin peptidase	Membrane	0,73	0,02	1446886
90	15901679	SP_1851	Uncharacterized protein	Cytoplasmic	0,41	0,05	1446897
91	15901684	SP_1856	Transcriptional regulator, MerR family	Cytoplasmic	0,58	0,04	38853
92	15901685	SP_1857	Cation efflux system protein	Membrane	1,44	0	1446899
93	15901688	SP_1860	Choline transporter	Membrane	0,53	0,04	38854
94	15901689	SP_1861	Choline transporter	Cytoplasmic	0,57	0,03	38854
95	15901690	SP_1862	Uncharacterized protein	Cytoplasmic	0,4	0,04	38854
96	15901691	SP_1863	Transcriptional regulator, MarR family	Cytoplasmic	0,49	0,02	38854
97	15901697	SP_1869	Iron-compound ABC transporter, permease protein	Membrane	1,5	0,01	38857
98	15901698	SP_1870	Iron-compound ABC transporter, permease protein	Membrane	1,49	0,01	38857
99	15901699	SP_1871	Iron-compound ABC transporter, ATP-binding protein	Cytoplasmic	1,51	0,02	38857

100	15901700	SP_1872	Iron-compound ABC transporter, iron-compound-binding protein	Lipoprotein	1,01	0,04	1446903
101	15901732	SP_1906	60 kDa chaperonin	Cytoplasmic	0,81	0,02	38862
102	15901733	SP_1907	10 kDa chaperonin	Cytoplasmic	0,75	0,03	38862
103	15901764	SP_1940	Protein RecA	Cytoplasmic	0,4	0,05	38871
104	15901824	SP_2001	Putative sensor histidine kinase	Membrane	0,39	0,02	38884
105	15901825	SP_2002	Uncharacterized protein	Membrane	0,45	0,02	38884
106	15901835	SP_2012	Glyceraldehyde-3-phosphate dehydrogenase	Cytoplasmic	0,77	0,03	1446953
107	15901888	SP_2069	Glutamate--tRNA ligase	Cytoplasmic	0,34	0,02	38895
108	15901889	SP_2070	Glucose-6-phosphate isomerase	Cytoplasmic	0,64	0,02	38896
109	15901890	SP_2071	Uncharacterized protein	Cytoplasmic	0,74	0,02	38896
110	15901984	SP_2174	D-alanine--poly(phosphoribitol) ligase subunit 2	Cytoplasmic	0,66	0,02	38917
111	15901985	SP_2175	DltB protein	Membrane	0,64	0,02	38917
112	15901986	SP_2176	D-alanine--poly(phosphoribitol) ligase subunit 1	Membrane	0,69	0,03	38917
113	15901993	SP_2186	Glycerol kinase	Cytoplasmic	1,78	0,02	38918
114	15901994	SP_2187	Conserved domain protein	Cytoplasmic	0,7	0,03	1447010
115	15902003	SP_2196	ABC transporter, ATP-binding protein	Cytoplasmic	0,61	0,01	38922
116	15902004	SP_2197	Putative ABC transporter, substrate-binding protein	Lipoprotein	0,58	0,03	38922
117	15902042	SP_2239	Serine protease	Membrane	1,64	0,01	38930
118	15902043	SP_2240	SpspoJ protein	Cytoplasmic	1,49	0,02	38930
119	330689322	nanA	Sialidase A	Unknown	-0.82	0,01	
120	15899952	SP_0003	Uncharacterized protein	Cytoplasmic	-0.81	0,01	38465
121	15899953	SP_0004	Ribosome-binding ATPase YchF	Cytoplasmic	-0.49	0,02	38466
122	15899966	SP_0018	Uncharacterized protein	Cytoplasmic	-0.64	0,02	1446345
123	15899968	SP_0020	tRNA-specific adenosine deaminase	Cytoplasmic	-0.87	0	1446347
124	15899969	SP_0021	Putative deoxyuridine 5'triphosphate nucleotidohydrolase	Cytoplasmic	-0.54	0,02	38468
125	15899970	SP_0022	Uncharacterized protein	Cytoplasmic	-0.49	0,02	38468
126	15899971	SP_0024	Uncharacterized protein	Cytoplasmic	-0.59	0,02	38469
127	15899972	SP_0025	Uncharacterized protein	Membrane	-0.67	0,03	38469
128	15899973	SP_0026	Uncharacterized protein	Membrane	-0.88	0,02	38469
129	15899987	SP_0042	Transport/processing ATP-binding protein ComA	Membrane	-0.60	0,02	38472
130	15899988	SP_0043	Transport protein ComB	Membrane	-0.78	0,01	38472
131	15899989	SP_0044	Phosphoribosylaminoimidazole-succinocarboxamide synthase	Cytoplasmic	-0.88	0,02	1446354
132	15899995	SP_0050	Bifunctional purine biosynthesis protein PurH [Includes: Phosphoribosylaminoimidazolecarboxamide formyltransferase	Cytoplasmic	-0.54	0,01	38475
133	15899996	SP_0051	Phosphoribosylamine--glycine ligase	Cytoplasmic	-0.73	0,01	1446355
134	15899998	SP_0053	N5-carboxyaminoimidazole ribonucleotide mutase	Cytoplasmic	-0.52	0,02	38476
135	15899999	SP_0054	N5-carboxyaminoimidazole ribonucleotide synthase	Cytoplasmic	-0.68	0,02	38476
136	15900000	SP_0055	Uncharacterized protein	Cytoplasmic	-0.49	0,05	38476
137	15900020	SP_0075	Phosphorylase, Pnp/Udp family	Cytoplasmic	-0.56	0,02	1446367
138	15900044	SP_0101	Putative transporter	Membrane	-0.61	0,05	1446380
139	15900060	SP_0118	tRNA-specific 2-thiouridylase MnmA	Cytoplasmic	-0.72	0	1446389
140	15900061	SP_0119	MutT/nudix family protein	Cytoplasmic	-0.81	0,01	38486
141	15900062	SP_0120	tRNA uridine 5-carboxymethylaminomethyl modification enzyme MnmG	Cytoplasmic	-0.71	0,02	38486
142	15900084	SP_0146	Uncharacterized protein	Membrane	-0.28	0,03	38494
143	15900089	SP_0151	Methionine import ATP-binding protein MetN	Cytoplasmic	-0.31	0,05	38495
144	15900090	SP_0152	Putative ABC transporter, permease protein	Membrane	-0.46	0,02	38495
145	15900101	SP_0163	Putative transcriptional regulator PlcR	Cytoplasmic	-0.45	0,03	1446401
146	15900102	SP_0164	Uncharacterized protein	Membrane	-0.97	0	38498
147	15900103	SP_0165	Flavoprotein	Cytoplasmic	-0.58	0,02	38498
148	15900156	SP_0220	50S ribosomal protein L24	Cytoplasmic	-0.45	0,03	38510
149	15900157	SP_0221	50S ribosomal protein L5	Cytoplasmic	-0.39	0,04	38510
150	15900158	SP_0222	30S ribosomal protein S14	Cytoplasmic	-0.37	0,05	38510
151	15900161	SP_0225	50S ribosomal protein L6	Cytoplasmic	-0.40	0,03	38511
152	15900176	SP_0240	Phosphoglycerate mutase family protein	Cytoplasmic	-0.35	0,04	1446413
153	15900201	SP_0267	Putative oxidoreductase	Cytoplasmic	-0.64	0,01	1446420
154	15900221	SP_0287	Xanthine/uracil permease family protein	Membrane	-1.03	0,01	1446429
155	15900222	SP_0288	Uncharacterized protein	Membrane	-0.98	0	1446430
156	15900246	SP_0313	Glutathione peroxidase	Cytoplasmic	-0.36	0,03	1446444
157	15900250	SP_0318	Carbohydrate kinase, PfkB family	Cytoplasmic	-0.73	0,02	38528
158	15900253	SP_0321	PTS system, IIA component	Cytoplasmic	-1.23	0,01	38529
159	15900254	SP_0322	Glucuronyl hydrolase	Cytoplasmic	-0.84	0,01	38529
160	15900255	SP_0323	PTS system, IIB component	Cytoplasmic	-0.68	0,03	38529
161	15900256	SP_0324	PTS system, IIC component	Membrane	-0.99	0,02	38529
162	15900257	SP_0325	PTS system, IID component	Membrane	-0.88	0,01	38529
163	15900258	SP_0326	Preprotein translocase, YajC subunit	Membrane	-0.85	0,01	38529
164	15900269	SP_0338	Putative ATP-dependent Clp protease, ATP-binding subunit	Cytoplasmic	-1.13	0,05	1446450
165	15900281	SP_0352	Capsular polysaccharide biosynthesis protein Cps4G	Cytoplasmic	-0.67	0,02	38533
166	15900283	SP_0354	Putative membrane protein	Membrane	-0.53	0,02	38533
167	15900290	SP_0366	Oligopeptide-binding protein AliA	Lipoprotein	-0.62	0,02	1446456
168	15900327	SP_0408	Sodium:alanine symporter family protein	Membrane	-0.58	0,01	1446466
169	15900331	SP_0412	Uncharacterized protein	Cytoplasmic	-0.64	0,01	38546
170	15900332	SP_0413	Aspartokinase	Cytoplasmic	-0.59	0,01	38546
171	15900334	SP_0415	Enoyl-CoA hydratase/isomerase family protein	Cytoplasmic	-0.54	0,03	1446471
172	15900335	SP_0416	Transcriptional regulator, MarR family	Cytoplasmic	-0.55	0,01	38547
173	15900336	SP_0417	3-oxoacyl-[acyl-carrier-protein] synthase 3	Cytoplasmic	-0.64	0,01	38547
174	15900338	SP_0419	enoyl-(acyl-carrier-protein) reductase	Cytoplasmic	-1.07	0,01	38548
175	15900339	SP_0420	Malonyl CoA-acyl carrier protein transacylase	Cytoplasmic	-1.22	0,01	38548
176	15900340	SP_0421	3-oxoacyl-[acyl-carrier protein] reductase	Cytoplasmic	-1.22	0,01	38548
177	15900341	SP_0422	3-oxoacyl-[acyl-carrier-protein] synthase 2	Cytoplasmic	-1.33	0,01	38548
178	15900342	SP_0423	Acetyl-CoA carboxylase, bitoin carboxyl carrier protein	Cytoplasmic	-1.33	0,01	38548
179	15900343	SP_0424	3-hydroxyacyl-[acyl-carrier-protein] dehydratase FabZ	Cytoplasmic	-1.41	0,01	38548
180	15900344	SP_0425	Acetyl-CoA carboxylase, biotin carboxylase	Cytoplasmic	-1.41	0,01	38548
181	15900345	SP_0426	Acetyl-coenzyme A carboxylase carboxyl transferase subunit beta	Cytoplasmic	-1.52	0,01	38548
182	15900346	SP_0427	Acetyl-coenzyme A carboxylase carboxyl transferase subunit alpha	Cytoplasmic	-1.62	0,01	38548
183	15900347	SP_0428	Uncharacterized protein	Cytoplasmic	-1.27	0,02	38548
184	15900348	SP_0429	Uncharacterized protein	Cytoplasmic	-0.86	0,04	38548
185	15900349	SP_0430	Uncharacterized protein	Membrane	-1.10	0,01	38548
186	15900350	SP_0431	Conserved domain protein	Membrane	-1.04	0,01	38548
187	15900368	SP_0451	Uncharacterized protein	Cytoplasmic	-0.41	0,03	1446479
188	15900380	SP_0464	Cell wall surface anchor family protein	Cell Wall	-0.53	0,02	38554
189	15900384	SP_0468	Putative sortase	Membrane	-0.62	0,01	38555
190	15900390	SP_0475	Uncharacterized protein	Cytoplasmic	-0.57	0,02	38557
191	15900391	SP_0476	PTS system, lactose-specific IIA component	Cytoplasmic	-0.88	0,02	38558
192	15900397	SP_0482	UPF0397 protein SP_0482	Membrane	-0.32	0,04	38560
193	15900410	SP_0496	Na/Pi cotransporter II-related protein	Membrane	-0.45	0,01	1446495
194	15900420	SP_0506	Integrase/recombinase, phage integrase family	Cytoplasmic	-0.37	0,04	1446501
195	15900440	SP_0526	Response regulator BlpR	Cytoplasmic	-0.38	0,02	38571
196	15900441	SP_0527	Putative sensor histidine kinase BlpH	Membrane	-0.44	0,02	38571
197	15900444	SP_0531	Bacteriocin BlpI	Secretory	-0.59	0,04	1446509
198	15900445	SP_0532	Bacteriocin BlpJ	Secretory	-0,6	0,02	1446510
199	15900447	SP_0534	Uncharacterized protein	Membrane	-0.54	0,03	1446512
200	15900457	SP_0544	Immunity protein BlpX	Membrane	-0.42	0,02	38576
201	15900460	SP_0547	Conserved domain protein	Membrane	-0.48	0,01	1446515

202	15900462	SP_0549	Uncharacterized protein	Cytoplasmic	-0.47	0,04	38577
203	15900514	SP_0606	Putative oxidoreductase	Membrane	-1.34	0	1446529
204	15900552	SP_0650	Uncharacterized protein	Membrane	-0.38	0,03	1446540
205	15900581	SP_0680	Pseudouridine synthase	Cytoplasmic	-0.75	0,04	1446550
206	15900600	SP_0701	Orotidine 5'-phosphate decarboxylase	Cytoplasmic	-0.85	0,01	38610
207	15900601	SP_0702	Orotate phosphoribosyltransferase	Cytoplasmic	-0.94	0	38610
208	15900626	SP_0729	Cation-transporting ATPase, E1-E2 family	Membrane	-0.43	0,03	1446559
209	15900632	SP_0737	Sodium-dependent transporter	Membrane	-0.73	0,02	38617
210	15900633	SP_0738	Uncharacterized protein	Cytoplasmic	-0.72	0,01	38617
211	15900637	SP_0742	DegV domain-containing protein SP_0742	Cytoplasmic	-1.23	0	38618
212	15900644	SP_0749	Branched-chain amino acid ABC transporter, amino acid-binding protein	Lipoprotein	-0.45	0,02	1446570
213	15900645	SP_0750	Branched-chain amino acid ABC transporter, permease protein	Membrane	-0.46	0,02	38620
214	15900646	SP_0751	Branched-chain amino acid ABC transporter, permease protein	Membrane	-0.44	0,01	38620
215	15900647	SP_0752	Branched-chain amino acid ABC transporter, ATP-binding protein	Cytoplasmic	-0.41	0,03	38620
216	15900648	SP_0753	Branched-chain amino acid ABC transporter, ATP-binding protein	Cytoplasmic	-0.43	0,02	38620
217	15900662	SP_0768	Probable dual-specificity RNA methyltransferase RlmN	Cytoplasmic	-0.39	0,03	38624
218	15900664	SP_0770	ABC transporter, ATP-binding protein	Cytoplasmic	-0.42	0,02	38624
219	15900678	SP_0785	Uncharacterized protein	Membrane	-0.53	0,02	38627
220	15900679	SP_0786	ABC transporter, ATP-binding protein	Cytoplasmic	-0.56	0,02	38627
221	15900680	SP_0787	Uncharacterized protein	Membrane	-0.50	0,02	38627
222	15900683	SP_0790	Conserved domain protein	Membrane	-0.58	0,02	38628
223	15900693	SP_0800	Uncharacterized protein	Membrane	-0.72	0	1446587
224	15900736	SP_0851	Glycerol-3-phosphate acyltransferase	Membrane	-0.64	0,01	1446606
225	15900756	SP_0873	Membrane protein	Membrane	-0.44	0,03	1446612
226	15900772	SP_0889	Uncharacterized protein	Cytoplasmic	-0.49	0,03	38647
227	15900796	SP_0916	Lysine decarboxylase	Cytoplasmic	-0.61	0,03	1446622
228	15900798	SP_0918	Polyamine aminopropyltransferase	Cytoplasmic	-0.64	0,01	38655
229	15900799	SP_0919	Uncharacterized protein	Cytoplasmic	-0.47	0,04	38655
230	15900800	SP_0920	Carboxynorspermidine decarboxylase	Cytoplasmic	-0.47	0,01	38655
231	15900801	SP_0921	Putative agmatine deiminase	Cytoplasmic	-0.57	0,02	38655
232	15900802	SP_0922	Carbon-nitrogen hydrolase family protein	Cytoplasmic	-0.82	0,03	38655
233	15900818	SP_0938	Ribosomal RNA small subunit methyltransferase I	Cytoplasmic	-0.50	0,01	38658
234	15900819	SP_0939	Uncharacterized protein	Cytoplasmic	-0.44	0,04	1446626
235	15900840	SP_0963	dihydroorotate dehydrogenase electron transfer subunit	Cytoplasmic	-0.76	0,01	38665
236	15900841	SP_0964	Dihydroorotate dehydrogenase 1B	Cytoplasmic	-0.83	0	38665
237	15900876	SP_1003	Uncharacterized protein	Membrane	-0.52	0,05	38673
238	15900878	SP_1006	Uncharacterized protein	Membrane	-0.49	0,03	1446639
239	15900885	SP_1013	Aspartate-semialdehyde dehydrogenase	Cytoplasmic	-0.38	0,05	38674
240	15900886	SP_1014	4-hydroxy-tetrahydrodipicolinate synthase	Cytoplasmic	-0.44	0,02	38674
241	15900910	SP_1039	Uncharacterized protein	Cytoplasmic	-0.50	0,02	1446653
242	15900928	SP_1058	Uncharacterized protein	Membrane	-1.41	0	1446664
243	15900929	SP_1059	Uncharacterized protein	Cytoplasmic	-1.09	0	1446665
244	15900930	SP_1060	Uncharacterized protein	Cytoplasmic	-0.54	0,02	38680
245	15900938	SP_1069	Uncharacterized protein	Secretory	-0.69	0,01	38682
246	15900939	SP_1070	Uncharacterized protein	Membrane	-0.62	0,01	38682
247	15900940	SP_1071	ABC transporter, ATP-binding protein	Cytoplasmic	-0.62	0,03	38682
248	15900948	SP_1079	GTPase ObgE	Cytoplasmic	-0.50	0,03	38684
249	15900949	SP_1080	Uncharacterized protein	Cytoplasmic	-0.48	0,04	38684
250	15900986	SP_1119	Glyceraldehyde-3-phosphate dehydrogenase, NADP-dependent	Cytoplasmic	-0.52	0,02	1446679
251	15901005	SP_1139	Uncharacterized protein	Cytoplasmic	-0.45	0,05	1446688
252	15901008	SP_1142	Uncharacterized protein	Cytoplasmic	-0.57	0,01	38696
253	15901091	SP_1229	Formate--tetrahydrofolate ligase	Cytoplasmic	-0.63	0,01	1446723
254	15901135	SP_1275	Carbamoyl-phosphate synthase large chain	Cytoplasmic	-0.69	0,02	1446736
255	15901136	SP_1276	Carbamoyl-phosphate synthase small chain	Cytoplasmic	-0.75	0,03	38726
256	15901137	SP_1277	Aspartate carbamoyltransferase	Cytoplasmic	-0.81	0,03	38726
257	15901138	SP_1278	Bifunctional protein PyrR [Includes: Pyrimidine operon regulatory protein; Uracil phosphoribosyltransferase	Cytoplasmic	-0.79	0,04	38726
258	15901154	SP_1294	Putative fluoride ion transporter CrcB 1	Membrane	-0.53	0,03	38731
259	15901155	SP_1295	Putative fluoride ion transporter CrcB 2	Membrane	-0.46	0,02	38731
260	15901245	SP_1391	Uncharacterized protein	Membrane	-0.42	0,05	38749
261	15901247	SP_1393	Uncharacterized protein	Cytoplasmic	-0.45	0,03	1446767
262	15901248	SP_1394	Amino acid ABC transporter, amino acid-binding protein	Lipoprotein	-0.46	0,02	1446768
263	15901256	SP_1402	NOL1/NOP2/sun family protein	Cytoplasmic	-0.51	0,04	38751
264	15901258	SP_1404	UPF0223 protein SP_1404	Cytoplasmic	-0.42	0,03	38751
265	15901259	SP_1405	Regulatory protein Spx	Cytoplasmic	-0.72	0,01	38751
266	15901280	SP_1427	Peptidase, U32 family	Cytoplasmic	-0.95	0,01	1446775
267	15901281	SP_1428	Uncharacterized protein	Cytoplasmic	-0.85	0,01	38757
268	15901282	SP_1429	Peptidase, U32 family	Cytoplasmic	-0.79	0,01	38757
269	15901285	SP_1433	Transcriptional regulator, AraC family	Cytoplasmic	-0.57	0,02	1446778
270	15901309	SP_1459	Uncharacterized protein	Membrane	-0.60	0,03	1446786
271	15901310	SP_1460	Amino acid ABC transporter, ATP-binding protein	Cytoplasmic	-0.97	0,01	38763
272	15901311	SP_1461	Amino acid ABC transporter, permease protein	Membrane	-0.86	0,02	38763
273	15901312	SP_1462	Uncharacterized protein	Cytoplasmic	-0.94	0,01	38764
274	15901313	SP_1463	Methylated-DNA--protein-cysteine S-methyltransferase	Cytoplasmic	-1.08	0,02	38764
275	15901314	SP_1464	Acetyltransferase, GNAT family	Cytoplasmic	-1.01	0,02	38764
276	15901315	SP_1465	Uncharacterized protein	Cytoplasmic	-2.10	0,01	38765
277	15901316	SP_1466	Hemolysin	Membrane	-2.13	0,01	38765
278	15901368	SP_1522	Conserved domain protein	Cytoplasmic	-0.38	0,02	38778
279	15901369	SP_1523	Snf2 family protein	Cytoplasmic	-0.41	0,02	38778
280	15901429	SP_1587	Oxalate:formate antiporter	Membrane	-0.90	0	1446821
281	15901440	SP_1600	Putative membrane protein	Membrane	-0.59	0,02	1446825
282	15901441	SP_1601	Uncharacterized protein	Membrane	-0.49	0,04	38797
283	15901450	SP_1610	SAM-dependent methyltransferase	Cytoplasmic	-0.38	0,02	38800
284	15901459	SP_1623	Probable cation-transporting ATPase exp7	Membrane	-0.50	0,04	1446830
285	15901470	SP_1634	Uncharacterized protein	Cytoplasmic	-0.36	0,04	1446839
286	15901480	SP_1644	D-aminoacyl-tRNA deacylase	Cytoplasmic	-0.38	0,04	38804
287	15901521	SP_1686	Uncharacterized oxidoreductase SP_1686	Cytoplasmic	-1.08	0	38815
288	15901522	SP_1687	Sialidase B	Membrane	-0.85	0,01	38815
289	15901523	SP_1688	ABC transporter, permease protein	Membrane	-0.95	0,01	38815
290	15901524	SP_1689	ABC transporter, permease protein	Membrane	-0.84	0,01	38815
291	15901557	SP_1724	Sucrose-6-phosphate hydrolase	Cytoplasmic	-0.49	0,03	38822
292	15901558	SP_1725	Sucrose operon repressor	Cytoplasmic	-0.54	0,03	38822
293	15901569	SP_1737	DNA-directed RNA polymerase subunit omega	Cytoplasmic	-0.54	0,04	38827
294	15901638	SP_1809	Transcriptional regulator	Cytoplasmic	-0.57	0,01	38840
295	15901639	SP_1810	Uncharacterized protein	Cytoplasmic	-0.72	0,01	38840
296	15901650	SP_1821	Sugar-binding transcriptional regulator, LacI family	Cytoplasmic	-0.52	0,02	1446888
297	15901721	SP_1894	Sucrose phosphorylase	Cytoplasmic	-0.65	0,02	1446911
298	15901725	SP_1898	Alpha-galactosidase	Cytoplasmic	-0.54	0,05	1446912
299	15901744	SP_1920	Transcriptional regulator, MarR family	Cytoplasmic	-0.56	0,03	38867
300	15901746	SP_1922	Probable transcriptional regulatory protein SP_1922	Cytoplasmic	-0.84	0,01	38867
301	15901747	SP_1923	Pneumolysin	Cytoplasmic	-1.37	0,02	38868
302	15901748	SP_1924	Uncharacterized protein	Cytoplasmic	-1.29	0,02	38868
303	15901749	SP_1925	Uncharacterized protein	Cytoplasmic	-1.19	0,02	38868

304	15901750	SP_1926	Uncharacterized protein	Membrane	-1.04	0,02	38868
305	15901766	SP_1942	Putative transcriptional regulator	Secretory	-0.29	0,05	38872
306	15901770	SP_1946	Putative transcriptional regulator PlcR	Cytoplasmic	-0.58	0,02	1446927
307	15901772	SP_1948	Conserved domain protein	Cytoplasmic	-0.76	0,04	38873
308	15901773	SP_1949	Uncharacterized protein	Cytoplasmic	-0.75	0,03	38873
309	15901774	SP_1950	Putative bacteriocin formation protein	Cytoplasmic	-0.51	0,03	1446929
310	15901775	SP_1951	Uncharacterized protein	Cytoplasmic	-0.61	0,04	38874
311	15901776	SP_1952	Uncharacterized protein	Membrane	-0.71	0,03	38874
312	15901777	SP_1953	Toxin secretion ABC transporter, ATP-binding/permease protein	Membrane	-0.70	0,01	38874
313	118090034	SP_1954	Serine protease, subtilase family	Secretory	-0.54	0,02	38874
314	15901782	SP_1959	Nucleoside diphosphate kinase	Cytoplasmic	-0.46	0,02	1446931
315	15901785	SP_1962	Uncharacterized protein	Cytoplasmic	-1.42	0	1446932
316	15901834	SP_2011	Pseudouridine synthase	Cytoplasmic	-0.43	0,03	1446952
317	15901849	SP_2028	Phosphotyrosine protein phosphatase	Membrane	-0.48	0,03	38887
318	15901862	SP_2041	Membrane protein insertase YidC 2	Membrane	-0.39	0,05	38890
319	15901874	SP_2054	Uncharacterized protein	Cytoplasmic	-0.67	0,02	1446967
320	15901880	SP_2060	Pyrrolidone-carboxylate peptidase 2	Cytoplasmic	-0.37	0,04	1446973
321	118090038	SP_2063	LysM domain protein	Membrane	-0.77	0,04	1446338
322	15901886	SP_2067	Uncharacterized protein	Cytoplasmic	-0.59	0,01	1446975
323	15901894	SP_2077	Arginine repressor	Cytoplasmic	-0.60	0,01	1446978
324	15901900	SP_2084	Phosphate-binding protein PstS 2	Lipoprotein	-1.06	0,01	1446982
325	15901901	SP_2085	Phosphate ABC transporter, permease protein	Membrane	-0.78	0,02	38898
326	15901902	SP_2086	Phosphate ABC transporter, permease protein	Membrane	-0.74	0,02	38898
327	15901903	SP_2087	Phosphate import ATP-binding protein PstB 3	Cytoplasmic	-0.87	0,01	38898
328	15901904	SP_2088	Phosphate-specific transport system accessory protein PhoU homolog	Cytoplasmic	-0.93	0,01	38898
329	15901930	SP_2115	Uncharacterized protein	Membrane	-0.51	0,02	38906
330	15901932	SP_2117	Uncharacterized protein	Membrane	-0.48	0,02	38906
331	15901933	SP_2118	Uncharacterized protein	Membrane	-0.45	0,03	38907
332	15901934	SP_2119	Putative transcriptional regulator	Cytoplasmic	-0.60	0,02	38907
333	15901947	SP_2133	Conserved domain protein	Cytoplasmic	-0.58	0,01	38909
334	15902021	SP_2217	Putative rod shpae-determining protein MreD	Membrane	-0.31	0,05	38926
335	15902036	SP_2233	Uncharacterized protein	Cytoplasmic	-1.84	0	1447021
336	15902038	SP_2235	Response regulator ComE	Cytoplasmic	-0.98	0	38929
337	15902039	SP_2236	Putative sensor histidine kinase ComD	Membrane	-0.64	0,01	38929
338	15902041	SP_2238	Ribosomal RNA large subunit methyltransferase H	Cytoplasmic	-0.42	0,01	1447023

Log2 fold changes (FC) are the ratio between deferoxamine treatment and control. Positive values indicate up-regulation, and negative values indicate down-regulation
